# *Plasmodium vivax* genetic diversity and heterozygosity in blood samples and resulting oocysts at the Thai–Myanmar border

**DOI:** 10.1186/s12936-017-2002-x

**Published:** 2017-09-05

**Authors:** Ingfar Soontarawirat, Chiara Andolina, Richard Paul, Nicholas P. J. Day, Francois Nosten, Charles J. Woodrow, Mallika Imwong

**Affiliations:** 1 Department of Clinical Tropical Medicine, Faculty of Tropical Medicine, Mahidol University, Bangkok, Thailand; 2 Shoklo Malaria Research Unit, Mahidol-Oxford Tropical Medicine Research Unit, Faculty of Tropical Medicine, Mahidol University, Mae Sot, Thailand; 3 Centre for Tropical Medicine and Global Health, Nuffield Department of Medicine Research Building, University of Oxford, Old Road Campus, Oxford, UK; 4 Unité de Génétique Fonctionnelle Des Maladies Infectieuses, Institut Pasteur, 28 rue du Docteur Roux, 75724 Paris, France; 5 Centre National de la Recherche Scientifique, URA3012, 28 rue du Docteur Roux, 75724 Paris, France; 6 Mahidol Oxford Research Unit, Faculty of Tropical Medicine, Mahidol University, Bangkok, Thailand; 7 Department of Molecular Tropical Medicine and Genetics, Faculty of Tropical Medicine, Mahidol University, Bangkok, Thailand

**Keywords:** Relapse, *Plasmodium vivax*, Oocyst, Meiosis, Genetic diversity

## Abstract

**Background:**

Polyclonal blood-stage infections of *Plasmodium vivax* are frequent even in low transmission settings, allowing meiotic recombination between heterologous parasites. Empirical data on meiotic products are however lacking. This study examined microsatellites in oocysts derived by membrane feeding of mosquitoes from blood-stage *P. vivax* infections at the Thai–Myanmar border.

**Methods:**

Blood samples from patients presenting with vivax malaria were fed to *Anopheles cracens* by membrane feeding and individual oocysts from midguts were obtained by dissection after 7 days. DNA was extracted from oocysts and parental blood samples and tested by microsatellite analysis.

**Results:**

A focused study of eight microsatellite markers was undertaken for nine blood stage infections from 2013, for which derived oocysts were studied in six cases. One or more alleles were successfully amplified for 131 oocysts, revealing high levels of allelic diversity in both blood and oocyst stages. Based on standard criteria for defining minor alleles, there was evidence of clear deviation from random mating (inbreeding) with relatively few heterozygous oocysts compared to variance across the entire oocyst population (F_IT_ = 0.89). The main explanation appeared to be natural compartmentalisation at mosquito (F_SC_ = 0.27) and human stages (F_CT_ = 0.68). One single human case produced a total of 431 successfully amplified loci (across 70 oocysts) that were homozygous and identical to parental alleles at all markers, indicating clonal infection and transmission. Heterozygous oocyst alleles were found at 15/176 (8.5%) successfully amplified loci in the other five cases. There was apparently reduced oocyst heterozygosity in individual oocysts compared to diversity within individual mosquitoes (F_IS_ = 0.55), but this may simply reflect the difficulty of detecting minor alleles in oocysts, given the high rate of amplification failure. Inclusion of minor allele peaks (irrespective of height) when matching peaks were found in related blood or oocyst samples, added 11 minor alleles for 9 oocysts, increasing the number of heterozygous loci to 26/176 (14.8%; p = 0.096).

**Conclusion:**

There was an apparently low level of heterozygous oocysts but this can be explained by a combination of factors: relatively low complexity of parental infection, natural compartmentalisation in humans and mosquitoes, and the methodological challenge of detecting minor alleles.

**Electronic supplementary material:**

The online version of this article (doi:10.1186/s12936-017-2002-x) contains supplementary material, which is available to authorized users.

## Background

*Plasmodium vivax* is the most widespread malarial species outside Africa, with up to 2.85 billion people living at risk of infection [1], several hundred million clinical cases occurring every year and consequent severe disease and death [2]. A significant problem for the elimination of *P. vivax* is its ability to form hypnozoite stages in the liver. These dormant stages result in intermittent relapses that can occur from 3 weeks up to several years after the initial infection [3], and according to the epidemiological setting a high proportion of patients can subsequently undergo relapse without primaquine treatment [4]. The relapse interval time differs substantially among *P*. *vivax* strains from short (weeks) in tropical areas with perennial transmission to long (months/years) in temperate zones with seasonal transmission [5]. The potential evolutionary advantage of relapses seems clear; in principle it provides increased opportunity for genetically distinct parasites to occur simultaneously within the blood and hence recombine in the mosquito during transmission. This would enable the generation of a relatively high degree of genetic diversity despite low transmission intensity [6, 7]. Consistent with this, the multiplicity of infection and genetic diversity are generally higher with *P. vivax* than equivalent measures in *Plasmodium falciparum* [8–10]. However there is also evidence for strong linkage disequilibrium [6, 11–13], suggesting that the recombination rate is low.

Whilst parasite population genetic structure can be inferred by genetic analysis of blood stage parasites that are relatively easy to sample, direct measures of recombination rates require analysis of oocysts, which contain the products of meiosis. A considerable number of studies of heterozygosity in oocysts in *P. falciparum* have been undertaken [14–20], but to date only one study of meiosis in *P. vivax* has been performed based on a single di-allelic marker [21]. This study was designed to measure the extent to which parasites recombine by studying the genotypes of oocysts and matching parental blood samples, and quantify the genetic diversity generated during transmission from monoclonal and polyclonal infections in man to mosquitoes.

## Methods

### Blood collection

5 ml of venous blood were withdrawn from patients attending the Shoklo Malaria Research Unit (SMRU) clinics on the Thailand–Myanmar border (Wang Pha, Mawker Thai, Mun Ru Chai, Mae Kong Khen and Mae La) whose blood film was positive for *P. vivax* gametocytes by microscopic examination. Gametocytaemia was expressed as number of gametocytes/500 white blood cells, assuming an average of 8000 WBC/µl [22].

### Mosquitoes rearing and blood feeding

*Anopheles dirus* B (*Anopheles cracens*) mosquitoes were reared under standard insectary conditions at 26–28 °C and 80% humidity in a secure insectary. 4–7 day old female mosquitoes were used for experiments. *Plasmodium vivax* infected blood samples were sent to the SMRU laboratories in Mae Sot. After plasma replacement with AB+ serum from a donor, blood samples were fed to 100 locally reared mosquitoes through a membrane feeding system (Haemotek^®^). Fully engorged mosquitoes were selected, moved to plastic containers and kept in incubators until dissection [23].

### Oocyst collection

Mosquito midguts were dissected on day 7 after membrane feeding. Midguts were stained with 1% solution of mercurochrome in phosphate buffer saline (PBS) to check them for the presence of oocysts and the number of oocysts per midgut was counted and recorded [24]. Infected midguts were stored in PBS at 4 °C until individual oocysts were isolated. Individual oocysts were isolated from each midgut under a stereo-microscope by using finely pointed glass capillaries made from 2 ml glass micro-pipettes elongated by heating over a Bunsen. Individual oocysts were kept in PBS total volume 100 µl and frozen at −80 °C until analysed.

### DNA extraction and genotyping

Genomic DNA was extracted from patient’s blood by QIAGEN DNA mini kit. Individual, frozen oocysts were thawed at room temperature before PCR. 8 polymorphic microsatellite markers: Pv3.27, Pv3.502, Pv8.504 [11] and MS5, MS6, MS7, MS8 and MS16 [25] were used to genotype the parasites of a subset of patients and corresponding individual oocysts following published protocols [11, 25]. The microsatellite PCR products were checked for quality and size against a standard DNA ladder (100 bp DNA ladder, New England Biolabs) under UV light and then sized relative to a Genescan 500 LIZ internal size standard that was performed by Macrogen Inc.. The microsatellite PCR products were separated on a capillary sequencer and scored using Peakscanner v1.0 software (Applied Biosystems). The expected finding was one or two alleles per oocyst (since the malaria oocyst stage is a direct result of a diploid stage); the presence of any additional alleles was interpreted as contamination. The presence of two or more alleles in amplified products from infected blood samples was interpreted as a multiple clone infection since the blood-stage malaria parasite is haploid (n). A locus was classed as having multiple alleles when the score of the minor peak was at least one-third the height of the predominant allele (major peak) [26] and when the number of repeat units between the two peaks was more than one repeat unit. Samples for which the PCR data were ambiguous (the required minimum peak height threshold was set as 300 fluorescent units) were re-amplified and the second result used. In addition a secondary analysis was undertaken in which minor allele peaks were included (irrespective of relative height) if there were matching peaks in blood or oocyst samples from the same original case.

### Infection rate and oocyst distribution

To characterize the distribution of *P. vivax* oocysts in the infected mosquitoes in different patients, the number of oocysts per mosquito was counted and the mean intensity and indices of parasite distribution within mosquitoes were computed by using the QUANTITATIVE PARASITOLOGY 3.0 [27].

### Genetic diversity

Genetic diversity was determined by the percentage of polymorphic loci and the haplotype diversity (expressed as ‘expected heterozygosity’ = H_exp_) (1 minus the sum of the squares of all allele frequencies) using GenAlex version 6.502 [28]. Multilocus linkage disequilibrium (LD) was calculated via a standardized index of association (I_A_) [29, 30]. This statistical test measures the extent of linkage equilibrium within a population by quantifying the amount of recombination among a set of haplotypes and detecting association between alleles at different loci. This statistical test was performed in the *poppr* R package using a permutation approach [31].

### Genetic structure

**Fig. 1:**
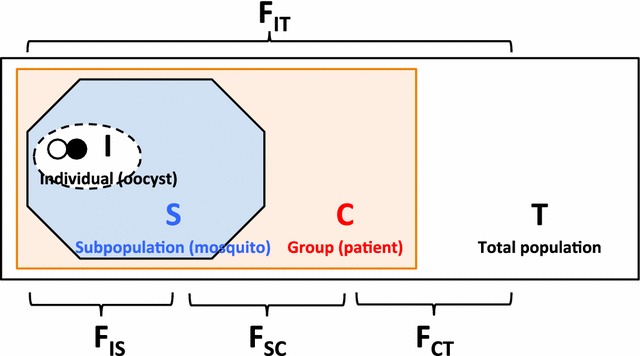
Overall scheme of populations and hierarchical F-statistics. See text and Appendix for further details

The population genetic structure of the oocysts was explored by calculation of coefficients of inbreeding (Wright’s F-statistics) that provide a measure of any deviation from expected diversity under random mating (Fig. 1) [32]. An inbreeding coefficient F = 0 equals random mating and F = 1 indicates complete selfing.

Oocyst genotypes can be naturally considered at four hierarchical levels: individual oocysts (I), subpopulations of oocysts in each mosquito (S), a smaller number of larger subpopulations of oocysts grouped by clinical case (C), and the entire set of oocysts (T). For each of these levels the average allelic diversity can be calculated (1 minus the sum of the squares of all allele frequencies within each population, averaged over all populations). In the case of individual oocysts this is the average heterozygosity. F-statistics are calculated as the difference between these levels of diversity, allowing assessment of the contribution of varying levels of population sub-structure to any overall deviation from random mating. Because there are four levels of organization there are three step-wise levels of possible inbreeding or compartmentalization (Fig. 1; Appendix).

These F-statistics were estimated by analysis of molecular variance (AMOVA) [33]. The significance of the fixation indices was tested using a non-parametric permutation approach, consisting of permuting haplotypes, individuals, or populations, among individuals, populations, or groups of populations.

## Results

### Blood samples and genetic diversity

Twenty-one patients presenting with febrile illness in 2013 were identified as having *P. vivax* infection by microscopic examination. Stored blood samples were later assessed for malaria species by PCR; all were confirmed to contain *P. vivax*. Four samples were also found to contain *P. falciparum*.

All blood samples were tested at two microsatellite markers to examine genetic diversity. At the 3.27 marker there were 14 different alleles (H_exp_ = 0.952) while at the 3.502 marker there were eight different alleles (H_exp_ = 0.843), indicating high genetic diversity at both loci. Eighteen samples had single genotypes at both loci; two had two genotypes at 3.27 and one sample had two genotypes at 3.502.

Eight microsatellite loci were studied in a subset of nine samples including the six cases in which oocysts were subsequently studied (see below). The number of distinct allelic variants for the eight loci in these blood samples varied from four to nine, with a mean ± SE H_exp_ of 0.87 ± 0.16 (Table 1). This indicated that these markers were suitable for examining parasite diversity in this setting.

**Table 1:** Diversity and expected heterozygosity at eight markers in nine blood samples

	Number of different alleles	Expected heterozygosity (H_e_)
Pv3.27	8	0.944
Pv3.502	6	0.833
Pv8.504	4	0.5
MS5	6	0.889
MS6	8	0.972
MS7	6	0.889
MS8	9	0.972
MS16	8	0.958
Mean	6.87	0.87
SD	1.64	0.16

### Oocyst studies

**Fig. 2:**
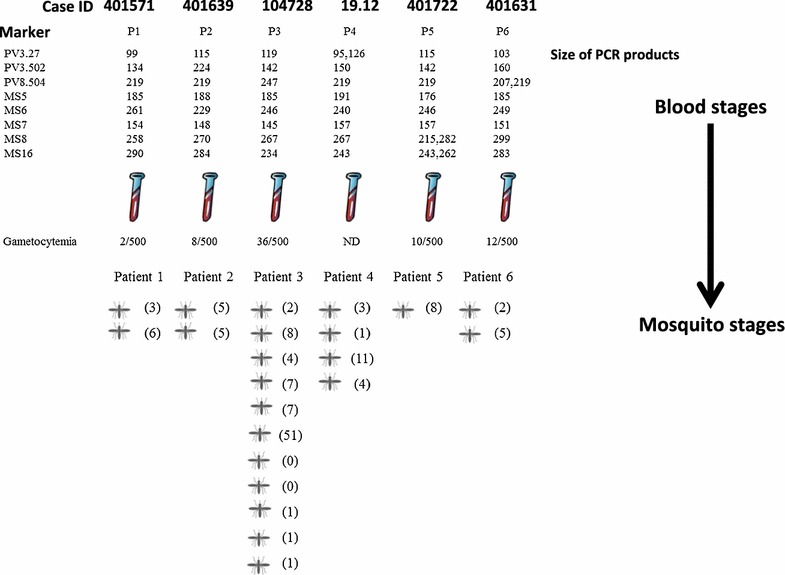
Study design and flow. The marker results for blood stages indicate that patients 1–3 appeared to be monoclonal infections and patients 4–6 polyclonal infections. The *bracketed numbers* next to each mosquito are the numbers of oocysts with at least one allele successfully amplified for each mosquito

Oocysts derived from six patients were studied; these were the first three patients determined to have single clone *P. vivax* blood infections and the first three patients determined to have multiple clone *P. vivax* infections, according to results from microsatellite analysis (see above and Fig. 2).

In these six cases, at least one locus was successfully amplified by PCR from 131 out of 203 oocysts, with 97 oocysts (74%) successfully amplified at three or more loci. Of the 131 oocysts with at least one locus successfully amplified, 97 oocysts were from infected mosquitoes fed on blood from the three patients judged initially to have monoclonal *P. vivax* infection and 34 oocysts were from the three cases judged to be polyclonal. Overall 607 loci out of a possible 1024 (57.9%) were successfully amplified. As seen with the blood stages, in the oocysts all microsatellite loci were highly polymorphic, with 5 to 8 alleles per locus (mean ± SE = 6.1 ± 0.9 alleles per locus) (Table 2). In the apparently monoclonal *P. vivax* infections, genetic diversity (H_exp_) ranged across loci from 0.44 to 0.67 (mean 0.61 ± SE 0.04) in blood samples and 0.078–0.36 (mean 0.18 ± SE 0.04) in corresponding oocysts. In the polyclonal *P. vivax* infections, H_exp_ ranged from 0.28 to 0.72 (mean 0.60 ± SE 0.06) in humans and 0.30–0.53 (mean 0.43 ± SE 0.07) in corresponding oocysts. The maximum number of alleles at a single locus within one mosquito was four.

**Table 2:** Genetic diversity of *P. vivax* in patient blood samples (P) and oocysts (O) for six selected cases

Measure	Infection	Sample	N	Locus								Mean	SE
				Pv3.27	Pv3.502	Pv8.504	MS5	MS6	MS7	MS8	MS16		
Number of alleles	Monoclonal	P	3	3	3	2	2	3	3	3	3	2.750	0.164
		O	97	4	3	2	2	3	3	3	3	2.875	0.227
	Polyclonal	P	3	4	3	2	3	3	2	4	3	3.000	0.267
		O	34	5	5	4	2	6	4	6	4	4.500	0.463
	All oocysts		131	7	6	5	5	6	6	8	6	6.125	0.927
Expected heterozygosity	Monoclonal	P	3	0.667	0.667	0.444	0.444	0.667	0.667	0.667	0.667	0.611	0.036
		O	97	0.290	0.187	0.177	0.069	0.360	0.078	0.159	0.119	0.180	0.036
	Polyclonal	P	3	0.722	0.667	0.278	0.667	0.667	0.444	0.722	0.611	0.597	0.055
		O	34	0.757	0.698	0.653	0.346	0.707	0.597	0.704	0.656	0.640	0.045
	All oocysts		131	0.527	0.354	0.436	0.301	0.474	0.406	0.433	0.492	0.428	0.069

Monoclonal and polyclonal infections are defined by initial blood stage results (see main text and Fig. 2)

### Genetic structure

Hierarchical F-statistics were obtained by AMOVA to infer oocyst population structure. There was evidence of compartmentalization both when comparing the sets of oocysts defined by individual patients to the entire oocyst population (F_CT_ = 0.68, p 0.000), and when comparing the subpopulations of oocysts in individual mosquitoes to the sets of oocysts derived from individual patients (F_SC_ = 0.27, p 0.000). In addition there was evidence of deviation from random mating (low heterozygosity) in individual oocysts compared to the subpopulations of oocysts in individual mosquitoes (F_IS_ = 0.55, p 0.000). These three levels of inbreeding combined to produce a very high level of overall inbreeding, with very low heterozygosity in oocysts relative to the population variance across the entire set of oocysts (F_IT_ = 0.89, p 0.000). There was no evidence of linkage disequilibrium in either population group (Table 3).

**Table 3:** Linkage disequilibrium of *P. vivax* oocyst haplotype at eight microsatellite loci, stratified by initial assessment of clonality in blood and by individual patient

Genotype	Patient	No. of oocyst	*I*_*A*_	*p* value
Polyclonal at 0 markers	401571	9	0.464	0.288
	401639	10	0.223	0.736
	104728	78	0.097	0.400
	All	97	0.22	0.661
Polyclonal at ≥1 marker	19.12	19	0.035	0.198
	401722	8	0.365	0.973
	401631	7	0.137	0.514
	All	34	0.241	0.717

*I*_*A*_ standardized index association

### Defining heterozygous and novel alleles in oocysts

One of the three infections judged as being single in the parental blood stage (patient 104728) generated 81 genetically identical oocysts (based on a total of 431 successfully genotyped loci); alleles were single and identical to the parent in every case. The remaining five cases yielded a total of 176 successfully amplified oocyst loci (for which raw peak heights are shown in Additional file 1). Fifteen (8.5%) of these oocyst loci were classed as heterozygous (10/118 from the three polyclonal parental blood infections and 5/58 from the two apparently single parental blood infections, p = 1.0) (Table 4). In all heterozygous oocysts only two alleles were found, providing no evidence for possible cross-contamination [34].

**Table 4:** Numbers and relationships of oocyst alleles

	3.27	3.502	8.504	MS5	MS6	MS7	MS8	MS16	All
Original criteria									
Single allele, same as blood	100	96	61	63	39	66	69	59	553
Single allele, different from blood	9	3	3	1	12	2	3	6	39
Two alleles, same as blood	2	0	0	0	1	0	1	0	4
Two alleles, one allele same as blood	2	1	1	0	3	0	0	1	7
Two alleles, both alleles different from blood	0	0	0	0	3	0	1	0	4
Modified criteria									
Single allele, same as blood	97	96	59	63	36	66	69	58	544
Single allele, different from blood	9	3	3	1	10	2	3	6	37
Two alleles, same as blood	2	0	0	0	3	0	1	1	7
Two alleles, one allele same as blood	5	1	2	0	6	0	0	1	15
Two alleles, both alleles different from blood	0	0	0	0	3	0	1	0	4

Total numbers of successfully amplified loci are shown, stratified according to relationship with parent blood samples. Modified criteria = inclusion of minor peaks on the basis of detection of matched peaks in related blood or oocyst samples

Of the 161 homozygous oocyst loci, 39 (24.2%) were novel i.e. not present in the parental blood sample (Table 4). These novel alleles were evenly distributed across the five cases; 13/53 (23.4%) homozygous oocyst alleles were novel from the two apparently single blood infections, compared to 26/108 (25.2%) homozygous oocyst alleles from the three polyclonal blood infections (p = 1.0).

The proportion of successfully amplified alleles that were novel compared to the parental sample varied according to the microsatellite locus, with MS6 and 3.27 yielding the highest proportions of oocysts with novel alleles (Table 4). There was a trend towards a positive correlation between these proportions and the allele diversity in blood stages for each locus, but this did not reach significance (Spearman r = 0.52, p = 0.19).

### Reassessment of minor alleles based on related samples

**Fig. 3:**
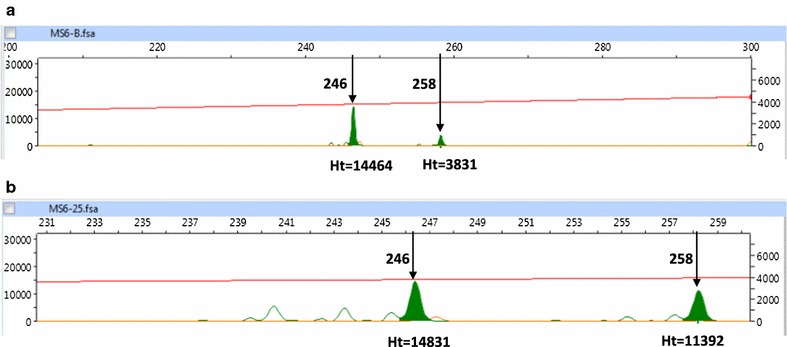
Defining minor alleles—an example. **a** Genescan trace for the PCR product derived from the blood sample for patient 401722, at the MS6 locus. The minor peak of length 258 has a height that is 26.5% the height of the major peak at 246. Under standard criteria this would not be considered a mixed infection. **b** Genescan trace for the PCR product derived from oocyst 1.6 for the same patient. The relative heights of the two peaks are similar indicating clearly a heterozygous oocyst. Given this new information the peak at 258 from the blood sample can now be considered genuine and hence the blood sample to contain a mixed infection

**Fig. 4:**
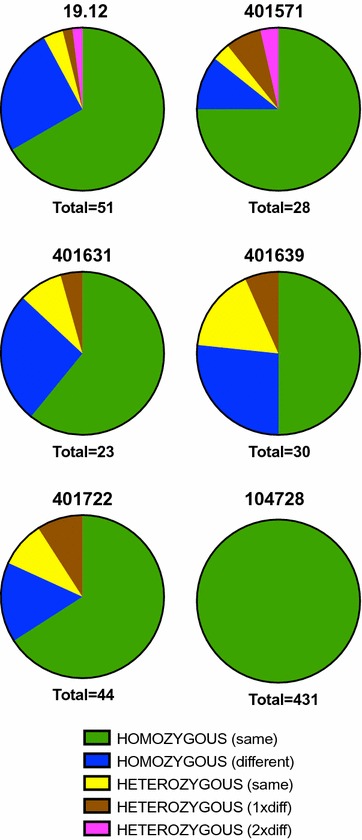
Homozygosity and relationship to blood stage infection for successfully amplified oocyst loci, stratified by human infection. Results are based on revised criteria involving detection of matched peaks in related blood or oocyst samples and are from all eight markers pooled. The three cases on the right were initially judged to be monoclonal in blood, but the presence of novel alleles in oocysts and use of the revised criteria for assessing minor alleles indicated that case 104728 was the only infection that was clonal at the blood stage (as well as in its oocysts)

As described above, using standard criteria for assessment of minor alleles, heterozygous oocyst alleles were found at 15/176 loci (discounting the entirely monoclonal infection). Examination of data from related samples identified a subset of minor peaks that were less than one-third the height of the main peak (and hence failed standard criteria) but present as significant alleles (major or minor peaks more than one-third of the height of a main peak) in related oocysts or blood samples (Fig. 3 shows an example). Assuming these to be genuinely heterozygous samples produced an additional 11 heterozygous alleles (within nine separate oocysts), and a total of 26/176 (14.7%) heterozygous alleles (p = 0.096 vs. standard criteria; Table 4). These were distributed among oocyst alleles derived from the three polyclonal parental blood infections (15/118 = 12.7%) and the two apparently monoclonal parental blood infections (11/58 = 19.0%, p = 0.27) (not including the globally clonal infection). The results of this retrospective assessment in terms of proportions of oocyst alleles that were homozygous (same as blood), homozygous (different from blood) or heterozygous (varying relationships to blood) are illustrated in Fig. 4.

The same approach also indicated that three blood-stage MS6 alleles (across different blood samples) initially judged homozygous were actually heterozygous. This included one of the blood samples initially considered monoclonal (case 401571).

## Discussion

Oocysts provide the only opportunity in the malaria life cycle to study the products of individual mating events that take place in the midgut of the mosquito [34]. The eight markers studied were highly polymorphic across different blood samples, even within a small sample size, with H_exp_ high and similar across all markers consistent with previous studies [6, 11–13, 35–41]. Initial measurements indicated that the average observed heterozygosity in oocysts (H_obs_ 0.03 ± 0.04) was much lower than the expected heterozygosity according to the diversity of the entire oocyst pool (H_exp_ 0.40 ± 0.07), leading to a very large value of F_IT_. This large discrepancy between observed and expected heterozygosities was approximately similar for each locus, indicating a considerable amount of inbreeding in the oocyst population. Several factors can explain these findings.

### High F-statistics (inbreeding)

There was good evidence for strong compartmentalization with a high level of inbreeding in oocysts grouped by human case relative to the entire population (F_CT_ = 0.68) and a somewhat lower level of inbreeding in oocyst subpopulations grouped by mosquito relative to people (F_SC_ = 0.27). These natural forces of compartmentalization are seen in many populations and are known as the Wahlund effect. Despite this, there was no evidence for LD, indicating that there was sufficient outbreeding to break down detectable LD (in the context of a small sample size) [15, 42, 43].

The third level of population differentiation, the inbreeding coefficient of individual oocysts relative to the subpopulation within each mosquito (F_IS_) was also substantial at 0.55, suggesting significant deviation from random mating and possible bias against outbreeding. Similar findings have been obtained in analogous studies of *P. falciparum* [14–20], but as also seen with *P. falciparum*, simpler explanations reflecting methodological issues are likely to explain much of the apparent inbreeding. Accurate detection of mixed alleles (and hence heterozygous infections) in oocysts is a substantial technical challenge given the low levels of DNA found within each oocyst, in the order of just tens of genome equivalents [16]. Although two alleles (if present) should be expected to be found in equal quantities in the oocyst, the template may be particularly low for some meiotic products because of unequal growth issues [18]; further there may be particular alleles that amplify with relatively low efficiency. These factors can combine to produce weak or absent signals (‘null’ alleles) [16]. In addition, a threshold has to be set to differentiate genuine minor peaks from false positive ones (resulting from PCR artefacts). This is normally set as a fixed proportion of the main peak (one-third here), but this approach clearly risks incorrectly excluding genuine alleles associated with lesser peaks [44]. Here an additional analysis was undertaken in which alleles less than one-third of the main peak height were included if the same sized peak was found in another oocyst sample and/or the parent blood sample. This led to a substantial rise in the number of heterozygous alleles (15–26) increasing the proportion of oocyst loci that were heterozygous to approximately 15%. Because the adjustment increases the number of heterozygous oocysts but would not affect the overall expected heterozygosity (because no new alleles enter that calculation), it would implicitly lead to a reduction in F_IS_, to a substantially lower level than 0.55. The same issue of heterozygote ‘deficit’ was carefully explored previously for *P. falciparum* with similar considerations applying [16].

### Defining the clonality of blood-stage infections

The clonality of blood-stage infections was initially assessed as usual by microsatellite typing of individual samples. However there were substantial numbers of downstream oocysts with novel alleles (not present in corresponding blood samples). In previous *P. falciparum* outcrossing experiments in monkey systems and in vitro, novel alleles were formed by recombination events during meiosis following cross-fertilization between two parasitic strains [45, 46]. In nature, more likely explanations include replication slippage, where the novel allele is one or a small number of repeats different to the parental genotype. However in one of the six blood infections studied, all the many downstream oocysts had identical alleles, suggesting that replication slippage during the sexual stage is rare.

A more likely explanation, particularly where the novel allele was found repeatedly in different oocysts and where size was too different to be accounted for by either intragenic recombination or replication slippage, is simply gametocyte alleles that failed to be amplified from the human sample because of loss through competitive amplification. Two of the three human cases assessed initially as being ‘monoclonal’ at the blood stage (cases 401571 and 401639) were associated with novel alleles coming up in oocysts (sometimes in heterozygous form) and can hence in retrospect be considered to have been polyclonal in terms of the parental blood stage. In summary the presence of new microsatellite alleles in oocysts (and salivary glands) of mosquitoes that feed on apparently mono-infected gametocyte samples indicates clones that were present, but not detected, in the gametocyte population and could infect mosquitoes [20].

In fact, several minor alleles in the blood samples themselves initially failed inclusion criteria (because of peak height) but were matched by downstream oocysts. One example was a minor peak in the blood sample for case 401571 coinciding with a clear peak in downstream oocysts, allowing a more direct re-assignment of this blood sample itself as polyclonal (at a single allele). Only one of the six cases studied in detail appears to have been truly clonal in the blood (case 104728).

This illustrates the potential for the microsatellite method to underestimate the polyclonality of parasites from blood-stage infections of *P. vivax* and *P. falciparum* [47, 48] with problems in detection of minority clones in blood samples reflecting suboptimal sensitivity of molecular methods and the numerical dominance of other clones [20, 49, 50]. One approach to this problem in *P. vivax* has been to clone PCR products into bacteria [44]. The process of studying oocysts carries similarities to cloning since it holds the potential to reveal individual blood genotypes, albeit following meiotic recombination and replication in oocysts.

## Limitations

The study was not without limitations. A relatively small number of human infections were studied because of logistical and resource implications. Furthermore, one-third of oocysts could not be genotyped at any locus. For the 131 oocysts in which at least one locus was successfully amplified, more than 600 oocyst loci were studied, an overall rate of successful amplification of around 60%. These figures seem broadly consistent with previous work on *P. falciparum* oocysts [18]. This is likely to have been associated with an increased chance of null alleles being present, thereby complicating interpretation of population statistics.

Novel approaches to population genetic studies using next-generation sequencing methods illustrate how PCR can underestimate the presence of minority clones, and are beginning to provide a deeper understanding of *P. vivax* biology across both global [51, 52] and local scales [53]. Selective whole genome amplification might offer the potential to carry out such studies on samples such as oocysts with relatively low quantities of DNA [54].

## Conclusions

In conclusion, the study provides more detailed information on the transmission genetics of *P. vivax*. The overall proportion of heterozygous oocyst alleles at first sight appeared surprisingly small. However, this appears to be explained in terms of parental samples with relatively low complexity of infection, natural compartmentalisation of infections (in people and mosquitos) and insensitive detection of minor alleles. Amended criteria based on the matching of potential minor peaks to those in related samples nearly doubled the sensitivity of heterozygous allele detection and also indicated reclassification of blood-stage infections as polyclonal. Taking into account all these factors, oocyst heterozygosity, and hence the potential for recombination between different alleles, appears to be at broadly the expected level.

## Appendix: Definitions of F-statistics

- F_IS_ = the heterozygosity of individual oocysts (I) compared to oocyst diversity in each individual mosquito subpopulation (S).
- F_IS_ is the inbreeding coefficient of individuals (I) relative to mosquitoes (S). It is also known as *f.* F_IS_ addresses the following question: given the range of genotypes encountered in all the oocysts from this mosquito, is the proportion of oocysts that are heterozygous as expected?
- F_SC_ = allelic diversity in oocysts from each individual mosquito subpopulation (S) compared to that in oocysts resulting from each individual patient sample (C).
  F_SC_ is the inbreeding coefficient of mosquitos (S) relative to the patient (C). F_SC_ addresses the following question: given the range of genotypes encountered in all the oocysts from each clinical case, is the allelic diversity of oocysts in mosquitoes as expected?
- F_CT_ = allelic diversity in oocysts resulting from each individual patient sample (C) compared to that in the entire set of oocysts (T).
  F_CT_ is the inbreeding coefficient in people (C) relative to the entire population (T). F_CT_ addresses the following question: given the range of genotypes encountered in the entire set of all oocysts, is the allelic diversity of oocysts found in this clinical case as expected?
- F_IT_ = the heterozygosity of individual oocysts (I) compared to allelic diversity in the entire set of oocysts (T).
  F_IT_ is the inbreeding coefficient of an individual (I) relative to the total (T) population. This represents the overall sum of the above three inbreeding coefficients. F_IT_ addresses the following question: given the range of genotypes encountered in the entire set of oocysts, is the proportion of oocysts that are heterozygous as expected?

## Additional file

**Additional file 1. ** Raw peak heights found in individual oocysts obtained from the five cases in which more than one allele was found at least one locus (in either blood or oocyst stages).
